# Determination of Glycerophospholipids in Biological Material Using High-Performance Liquid Chromatography with Charged Aerosol Detector HPLC-CAD—A New Approach for Isolation and Quantification

**DOI:** 10.3390/molecules27103356

**Published:** 2022-05-23

**Authors:** Magdalena Rosłon, Małgorzata Jaworska, Elżbieta L. Anuszewska

**Affiliations:** Department of Biochemistry and Biopharmaceuticals, National Medicines Institute, Chełmska 30/34, 00-725 Warsaw, Poland; m.roslon@nil.gov.pl (M.R.); e.anuszewska@nil.gov.pl (E.L.A.)

**Keywords:** HPLC-CAD, glycerophospholipids, cell membranes

## Abstract

The method of using high-performance liquid chromatography with a charged aerosol detector method (HPLC-CAD) was developed for the separation and determination of phospholipids isolated from cell membranes. The established cell lines—normal and neoplastic prostate cells and normal skin fibroblasts and melanoma cells—were selected for the study. Chromatographic separation was performed in the diol stationary phase using a gradient elution based on a mixture of n-hexane, isopropanol and water with the addition of triethylamine and acetic acid as buffer additives. Taking the elements of the Folch and Bligh–Dyer methods, an improved procedure for lipid isolation from biological material was devised. Ultrasound-assisted extraction included three extraction steps and changed the composition of the extraction solvent, which led to higher recovery of the tested phospholipids. This method was validated by assessing the analytical range, precision, intermediate precision and accuracy. The analytical range was adjusted to the expected concentrations in cell extracts of various origins (from 40 µg/mL for PS up to 10 mg/mL for PC). Both precision and intermediate precision were at a similar level and ranged from 3.5% to 9.0%. The recovery for all determined phospholipids was found to be between 95% and 110%. The robustness of the method in terms of the use of equivalent columns was also confirmed. Due to the curvilinear response of CAD, the quantification was based on an internal standard method combined with a power function transformation of the normalized peak areas, allowing the linearization of the signal with an *R*^2^ greater than 0.996. The developed method was applied for the isolation and determination of glycerophospholipids from cell membranes, showing that the profile of the tested substances was characteristic of various types of cells. This method can be used to assess changes in metabolism between normal cells and neoplastic cells or cells with certain pathologies or genetic changes.

## 1. Introduction

Phospholipids belong to the class of complex lipids, the molecular skeleton of which is glycerol (glycerophospholipids) or the long-chain amino alcohol sphingosine (phosphosphingolipids) [1]. Glycerophospholipids (GPLs) are the most common phospholipids in nature. They are present in the membranes of all cell types, but the content of individual phospholipid classes depends on the type of tissue. The asymmetric distribution of phospholipids within the membranes constituting the cell structure is also characteristic. Phosphatidylcholine (PC) is present in the greatest amount (as much as 40–50% of all phospholipids), but it is mainly present in the outer layer of the cells. In the inner layer, phosphatidylethanolamine (PE, approximately 20–30% of all phospholipids) and phosphatidylserine (PS, approximately 5–10% of all phospholipids) predominate. Phosphatidylinositol (PI) makes up 1–10% of all phospholipids and is mainly found inside the cell. It is most often employed as a precursor to many PI phosphates, which are involved in various cell signaling processes [2].

The available literature describes a number of methods dedicated to the determination of phospholipids by using high-performance liquid chromatography (HPLC) mainly using so-called universal detectors [3,4,5,6,7]. The applicability of refractometric detection [8], due to the fact of its limited sensitivity and the necessity to use isocratic elution, is currently marginal. A breakthrough in the chromatography of lipids and phospholipids has taken place due to the development of detectors enabling the acquisition of nonvolatile analyte particles after the evaporation of the mobile phase. Apart from the evaporative light scattering detector (ELSD), a corona charged aerosol detector (CAD) was found to be suitable and, nowadays, is increasingly popular. Mass spectrometry is another method of choice, but its application, despite the possibility of identifying compounds in the orthogonal mode, requires specialized equipment, substantial technical resources and human skills, and its use is therefore not widespread.

CAD detection was first described in 2002 by Dixon and Peterson [9]. The detector uses the measurement of the charge adsorbed on the analyte particles, which is derived from the stream of ionized nitrogen molecules (N^2+^) generated during corona discharges. As a result of the ionized gas collision with the analyte particles, the charge is transferred to the substance assayed [10,11]. This method of “labeling” analyte particles “labeling” means that the signal in CAD detection depends primarily on the concentration of the determined compound and not on its structure. CAD detection is treated as equivalent to ELSD in terms of applicability; however, it usually offers wider analytical range and better sensitivity [3].

The literature shows that most of the HPLC-CAD systems proposed for lipid compound analysis are based primarily on silica [3,12,13,14] or, as a second choice, on diol [4,15,16] columns although, C8, C18 and HILIC stationary phases were also used [17,18,19]. Mobile phases composed of solvent mixtures, such as n-heptane, n-hexane, isopropanol, chloroform, methanol, iso-octane or methyl t-butyl ether [3,4,6,12,15], were usually selected. Among the described studies, CAD detection was used mainly for testing pharmaceuticals (lipid excipients, liposomes) [18,20] or food samples such as edible oils, eggs and dairy products [4,12,13,15,16]. Despite the increasing use of CAD, a limited number of reports concern lipid studies in biological material such as cells or tissues [3,19].

The aim of this study was to develop a chromatographic method for the separation and quantification of various classes of glycerophospholipids in cell membranes using HPLC with CAD detection. The assay of the GPL and, in particular, the analysis of the proportions between their classes could be used in the assessment of changes in metabolism between normal and neoplastic cells or in cells burdened with certain pathologies and genetic changes. Regarding the intended use of the method for testing biological material of various origin (cultured cells, blood cells, etc.), it was premised to obtain a wide analytical range and to determine even low concentrations of GPL. During the study, special effort was devoted to refining the procedure of lipid isolation employing ultrasound-assisted extraction (UAE). UAE uses ultrasound energy and solvents to extract target compounds from various matrices and belongs to the modern techniques of lipid isolation. Compared to other techniques, it provides the highest efficiency of lipid extraction [21,22], affecting the repeatability and comparability of the results.

## 2. Results and Discussion

### 2.1. Method Development

#### 2.1.1. Optimization of Phospholipid Extraction Conditions

Extraction conditions were optimized with LNCaP cells. Ultrasound-assisted extraction (UAE) was used to increase its efficiency. Each extraction step involved pulsed ultrasound driven mixing of organic and water layers to increase the surface of lipid exchange between phases (see Section 3.3). In contrary to other reports [22,23], the isolation of lipid fractions from cell membranes was carried out at low temperature due to the need to limit the activity of phospholipases and reduce the decomposition of phospholipids in the samples. Both the energy and time of sonication were not optimized, because other data indicate that in a few seconds, in small volumes, adequate phase dispersion occurs and cell membranes completely disintegrate [24]. Instead, we focused on solvent selection and the number of extraction steps as key points for efficiency increase. 

The experiments began with the use of one-fold extraction with the CHCl_3_:MeOH mixture (2:1 *v*/*v*) according to the Folch method [25]. When the number of extractions increased to three with the same mixture, a gradual increase in extraction yield was observed. However, a literature search revealed that the Folch extraction solvent may not be sufficient to completely isolate the most polar classes of phospholipids, namely, PS and PI. The authors Bligh and Dyer [26,27] proposed for this purpose a mixture of CHCl_3_:MeOH in a 1:1 (*v*/*v*) proportion. On this basis, in order to ensure efficient extraction of all phospholipid classes, both less and more polar, first and second extraction steps were performed with a mixture of CHCl_3_:MeOH (2:1 *v*/*v*), and then the third one was performed with the solvent recommended by Bligh and Dyer, i.e., CHCl_3_:MeOH (1:1 *v*/*v*). It was noted that the proposed procedure, compared to the three-fold extraction with the CHCl_3_:MeOH (2:1 *v*/*v*) mixture, provided unchanged recovery for PE and PC, while PS and PI recovery increased 1.7 and 2.0 times, respectively.

The data in the literature show that the content of individual phospholipids and the sum of phospholipids in biological material depend on the type of tissue. In mammalian cells, this value is in the range of 10–80 µg/10^6^ cells [28,29]. The content of PI is the lowest among the classes of phospholipids analyzed in this study, usually approximately 5–20 times less than the content of PC, which accounts for approximately 40–50% of all phospholipids present in cells [30,31,32]. Hence, the amount of biological material necessary to obtain the concentration of PE, PC, PS and PI, thereby enabling their determination using the developed method, was estimated and then confirmed experimentally using LNCaP and PNT1A cells. It was found that approximately 10–50 × 10^6^ cells were required for a single sample preparation.

#### 2.1.2. Optimization of Chromatographic Conditions

The properties of phospholipids, particularly their hydrophobicity, suggest the use of normal-phase chromatography as an optimal condition for their separation. Due to the presence of polar groups in phospholipid molecules (i.e., choline, serine, inositol and ethanolamine), the addition of buffering components to the mobile phase (e.g., formic acid or diethylamine) is advantageous [5,33].

To ensure optimal HPLC-CAD separation of the phospholipids isolated from cell membranes, a number of experiments were carried out using different chromatographic systems. Silica columns, YMC-Pack Sil-60 150 × 4.6 mm, 3 µm (YMC, Kyoto, Japan) and Luna Silica 250 × 4.6 mm, 5 µm (Phenomenex, Torrance, CA, USA) as well as diol stationary phases (i.e., Lichrosorb Diol 150 × 3.2 mm, 5 µm (Hichrom, Berkshire, UK) and LiChrospher Diol 250 × 4.0 mm, 5 µm (Merck, Darmstadt, Germany)), were selected. We attempted to separate phospholipids using different mobile phases. For this purpose, eluents based on mixtures of CHCl_3_:MeOH:H_2_O or Hex:IPA:H_2_O were used at various volume ratios with the addition of buffering agents such as diethylamine, triethylamine, triethanolamine, formic acid, acetic acid or ammonium formate. The buffering agents were added in amounts not exceeding 2% of the pure component in the mobile phase. During the gradient elution, the most hydrophobic solvent (i.e., CHCl_3_ or Hex) was reduced, and the amount of polar solvent (i.e., MeOH, IPA or water) was increased, keeping the miscibility of eluents A and B possible, while the content of buffering components remained unchanged.

It was found that mobile phases based on CHCl_3_:MeOH mixtures generated significantly lower signals of phospholipids and higher detector noise compared to the phases containing the Hex:IPA system. The reason for this effect is the lower volatility (higher boiling point) of chloroform than hexane, affecting the efficiency of evaporation of the mobile phase in a CAD detector, which operates at ambient temperature. Hence, we decided to continue with the Hex:IPA-based mobile phase. The addition of CH_3_COOH and TEA to the mobile phase was finally selected to ensure the adequate ionization of the compounds to be determined. Compared to other modifiers tested or proposed by different authors [5,33], CH_3_COOH and TEA had the lowest impact on the noise and baseline drift of the chromatogram, especially during the gradient elution. In the case of silica-packed columns, significant changes in the elution order of individual phospholipid fractions were observed, depending on the CH_3_COOH and TEA ratio in the mobile phase. On the other hand, the diol packing columns showed, under analogous conditions, smaller differences in selectivity and shorter retention times, while maintaining the appropriate resolution of the system. For this reason, a column with a spherical diol stationary phase, namely, LiChrospher Diol 250 × 4.0 mm, 5 µm, was selected for further research.

Based on the different polarities of lipids present in cell extracts, a gradient elution was considered to be the most suitable in order to achieve sufficient system selectivity. At the same time, due to the fact that the CAD detector shows a similar response for various compounds only under isocratic conditions [11], we attempted to develop the method so that the mobile phase composition during the elution of all of the phospholipids under investigation would not change (isocratic step at approximately 5–14 min of gradient elution). This would enable the determination of PE, PC, PS and PI with potentially one potential reference solution, e.g., PC.

The intended elution approach turned out to be unsuitable due to the increase in the width of the last eluting peaks (i.e., PS and PI), which resulted in a decrease in their height, thus limiting the method’s sensitivity. The common solution for CAD detection could be the use of mobile phase compensation, which requires additional mobile phases of the opposite composition to be mixed with column eluate just before the detector [34,35]. However, keeping in mind that this generates a double consumption of solvents, also causes the dilution of the eluted analyte zones and reduces the method’s sensitivity, this approach was not applied in the current study. It was decided to continue with gradient elution of GPL under interest and, hence, to calibrate with reference materials for all the analyzed phospholipids, respectively.

### 2.2. Identification of Phospholipid Peaks

In the chromatograms of the cell extracts, apart from the PE, PC, PS and PI peaks, signals from other phospholipid classes were also visible. We attempted to identify them by comparing the retention times with the chromatograms acquired for the reference material of phosphatidic acid (PA), phosphatidylglycerol (PG), cardiolipin (CL), sphingomyelin (SM), *lyso*−phosphatidylserine (LPS) and soy lecithin (containing a mixture of PE, PC, PI and LPC). 

**Figure 1:**
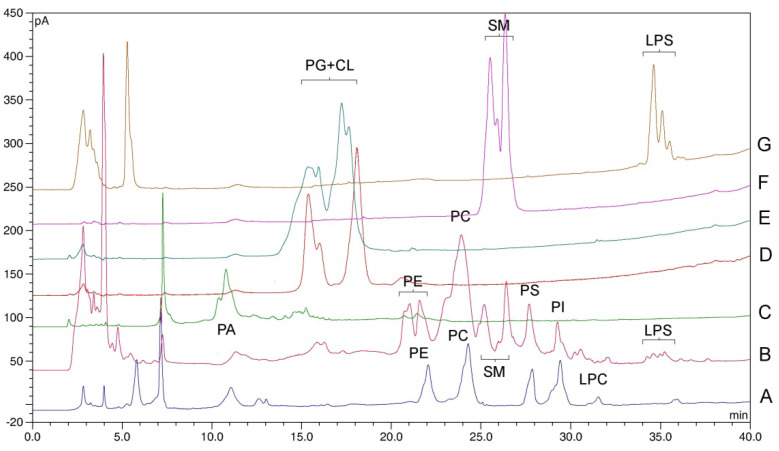
Chromatogram with the identification of phospholipid peaks extracted from cells. A—soy lecithin fortified with PS; B—human normal fibroblasts (BJ cells); C—phosphatidic acid (PA); D—cardiolipin (CL); E—phosphatidylglycerol (PG); F—sphingomyelin (SM); G—*lyso*−phosphatidylserine (LPS).

Phospholipids are a heterogeneous class of compounds due to the varied composition of fatty acids esterifying glycerol. As a result, GPL peaks may demonstrate polydispersity, depending on the proportion of fractions with different fatty acid chain lengths and the degree of saturation. For the same reason, phospholipids from different sources (plant and animal, tissue types) may show different peak shapes, and their retention time may vary slightly (Figure 1). Some of the peaks observed in the cell extracts were partially split (e.g., PE, SM or LPS) or tended to reveal not fully resolved subfractions (i.e., PC and PI). This was also observed by other authors [15,16,36]. In order to ensure correct identification, spiking with a given phospholipid was performed, selecting the reference material with possibly similar origin to the tested sample (see Section 3.1).

The results of the identification are presented in Figure 1. The chromatograms show that among the identified peaks, only PG and CL were eluted at the same time; however, the problem with the separation of these two substances has been reported [37]. The peaks visible in the initial part of the chromatogram (up to approximately 8 min), corresponding to nonpolar lipids, were not identified, as they were not the target of current study.

### 2.3. Methodology for Phospholipid Quantification

During the study, when continuous chromatography lasted up to 10–14 days, some instability in the CAD detector response was observed for the tested substances. Therefore, cholic acid was introduced as an internal standard, the elution of which did not interfere with the peaks of other analytes. Cholic acid was added to the solvent to dissolve the evaporated lipid extracts. The area of the phospholipid peaks was first divided by the internal standard area, and the resulting value (normalized peak area) was used for further calculations. The normalization of peak areas made it possible to compare the responses between the chromatograms recorded at different time points of the sequence, regardless of their length.

The split-to-baseline integration was used for all tested phospholipids. In the case of incompletely resolved GPL fractions, this approach yielded a meaningful result when the height ratio of the adjacent peaks was not less than 1:10 [38].

Due to the specificity of the CAD, the acquired signal only shows linearity in a narrow concentration range [39]. With the widened range, the relationship between the response and the concentrations is always curvilinear; thus, a quadratic, logarithmic or power fit is usually applicable [34,40,41,42]. In the current study, the following transformation was applied:

(1) $$A_{lin}=\frac{A^{X}}{scale^{\left(X-1\right)}}$$

where:

*A*—normalized peak area;
*X*—power function value;
*scale*—full-scale range of the detector (e.g., 500 pA);
*A_lin_*—response value eligible for linear fit calibration.

**Figure 2:**
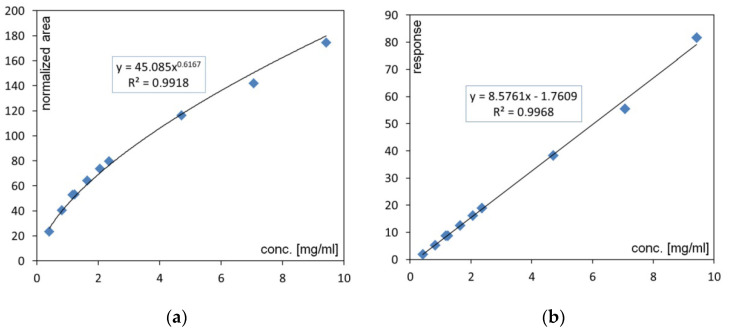
Example of (**a**) curvilinear relation between a normalized peak area and an analyte concentration (PE) and (**b**) the result of data transformation with Equation (1), leading to a linear calibration curve applied for assay (PE). The power function value was set at 1.85.

This transformation was applied in order to linearize the data and to use a common approach for standard curve linear calibration [43]. A result of data transformation and linearization is presented in Figure 2 as an example.

### 2.4. Method Validation

In order to test the robustness of this method, three other diol columns of the same size were tested: Lichrospher Diol of another batch (Merck, Darmstadt, Germany) and Lichrosorb Diol (Hichrom, Berkshire, UK) and Supelcosil LC-Fuji Diol (Supelco, Bellefonte, PA, USA). A 1 mg/mL soy lecithin solution enriched with PS at 0.2 mg/mL was injected onto each column and run with the same chromatographic conditions. Slight differences in resolution between the phospholipid peaks with unchanged selectivity were observed, which confirmed the stability of the method (Table 1). The Supelcosil LC-Fuji Diol column was considered unsuitable for the purpose defined in this study due to the highest baseline noise and the lowest signal for individual phospholipids. The other results indicate the possibility of using LiChrosorb Diol and LiChrospher Diol columns as alternatives.

The developed method for the determination of phospholipids in biological materials was validated for the following parameters: specificity, analytical range, precision, intermediate precision and accuracy (based on the recovery). The method was validated by applying the rules of the ICH Q2 (R1) guideline [44]. The validation results are presented in Table 2.

The specificity of the method was ensured by both the selectivity of the extraction and the selectivity of the chromatography. The extraction using a mixture of chloroform and methanol was dedicated to the isolation of lipid compounds from biological material [25,26]. In turn, the chromatographic conditions determined the elution of the neutral lipid fractions before the GPL fractions. Finally, the specificity of the method was confirmed at the peak-identification stage (Figure 1), showing no interference from other lipid components.

The evaluation of the analytical range of the method was carried out using solutions of reference materials in a wide concentration range for all the determined phospholipids as given in Table 2. The upper limit of the analytical range was set at the concentration at which the detector response had not yet reached a plateau. The lower limit of the analytical range was adjusted to the expected level of phospholipids in the biological material with the assumption that the sample availability may be limited (e.g., in the case of blood samples).

Although the ICH Q2 guideline does not formally require the determination of a detection limit (LOD) and quantification limit (LOQ) for the methods to be applied for assay [44], we attempted to determine the LOQ level according to mathematical assumptions based on the available data. The relationship between the signal-to-noise ratio (S/N) and the analyte concentration allowed the LOQ value to be calculated, which was 20 for PE, 3 for PC, 5 for PS and 9 μg/mL for PI.

Bearing in mind the target application of the developed method and the fact that the biological material contributes the most to results’ variability, the validation of the method in terms of precision, intermediate precision and recovery was carried out using extracts from PNT1A cells, which were prepared using the developed procedure of lipid isolation.

The precision of the method was assessed by determining the phospholipids of six independently prepared extracts from PNT1A cells at a density of 50 × 10^6^ cells/mL, and it is expressed as the relative standard deviation (RSD) of phospholipid content in the extracts. The intermediate precision was assessed by comparing the results of the precision study with those obtained for the next six extracts prepared independently by a second analyst on a different day, using a different set of reagents and reference materials. The groups of results obtained by both analysts were statistically analyzed for the equality of variance with the F-Snedecor test and the equality of means for unrelated groups. For all the phospholipids tested, it was shown that the results obtained by both analysts were characterized by the same variance and that the average of both groups did not differ significantly. The intermediate precision of the method was finally expressed as the RSD of the phospholipid content for 12 extracts. The RSD values of the obtained results, for both precision (n = 6) and intermediate precision (n = 12), were at a similar level and ranged from 3.5% to 9.0% for all analyzed substances. Thus, it can be concluded that the developed method gives reproducible results and is suitable for the intended purpose.

The accuracy of the method was tested by assessing the recovery. For this purpose, the suspensions of PNT1A cells at a density of 25–37.5 × 10^6^ cells/mL were fortified with individual phospholipids to the level determined in the cell suspensions at a density of 50 × 10^6^ cells/mL and extracted using the developed method. The recovery was calculated based on the difference in the content of individual phospholipids in the extracts between the fortified and nonfortified samples with respect to the theoretical number of analytes added to the cell suspension. The recovery for all of the determined phospholipids was at the appropriate level and ranged from 95% to 110% (Table 3). On the basis of the obtained validation results, the developed HPLC-CAD method was considered suitable for determination of PE, PC, PS and PI in the cell extracts.

### 2.5. Applicability of the Method

**Figure 3:**
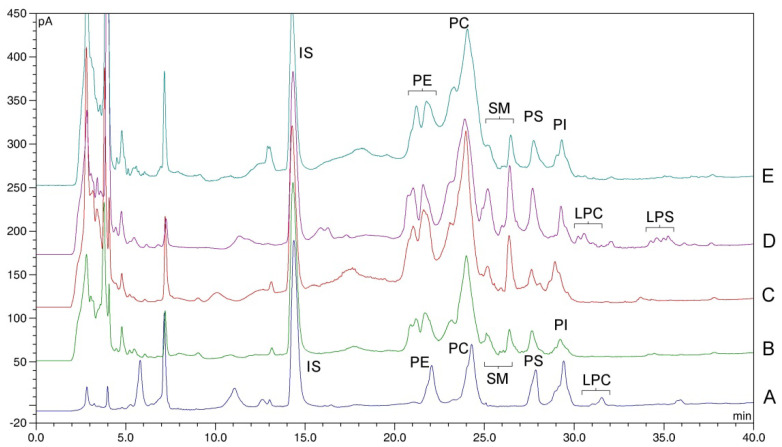
Separation of phospholipid fractions extracted from the cell lines used in the study, compared with soy lecithin fortified with PS: A—soy lecithin fortified with PS; B—human normal prostate cells (PNT1A); C—human neoplastic prostate cells (LNCaP); D—human normal fibroblasts (BJ); E—human melanoma cells (HTB 140).

The applicability of the method was verified on the two pairs of different types of cells (Figure 3):

- Human normal (PNT1A) and neoplastic (LNCaP) prostate cells;
- Human normal fibroblasts (BJ) and melanoma cells (HTB 140).

The results are presented in Table 3.

**Table 3:** Results of phospholipids assay for different cell lines. Asterisks indicate significant statistical difference between results for the neoplastic and respective normal cell lines: * *p* < 0.05; ** *p* < 0.01; *** *p* < 0.001.

	Cell Lines	PNT1A Normal Prostate Cells	LNCaP Neoplastic Prostate Cells	BJ Human Normal Fibroblasts	HTB 140 Human Melanoma Cells
Phospholipids					
Content		n = 5	n = 5	n = 4	n = 5
PE	Mean ± SD (nmol/mln cells)	12.7 ± 1.0	40.5 ± 5.0 ***	17.1 ± 1.8	23.6 ± 3.1 **
	mean (mol%) ^1^	48.2%	56.7% **	35.7%	33.9%
PC	Mean ± SD (nmol/mln cells)	10.8 ± 0.8	24.5 ± 1.8 ***	24.1 ± 1.0	40.4 ± 6.2 **
	mean (mol%) ^1^	41.1%	34.4% **	50.3%	57.6% *
PS	Mean ± SD (nmol/mln cells)	1.1 ± 0.1	1.9 ± 0.2 ***	3.3 ± 0.3	2.1 ± 0.2 ***
	mean (mol%) ^1^	4.3%	2.6% ***	6.9%	3.0% ***
PI	Mean ± SD (nmol/mln cells)	1.7 ± 0.2	4.5 ± 0.3 ***	3.4 ± 0.3	3.8 ± 0.6
	mean (mol%) ^1^	6.4%	6.3%	7.1%	5.5% *

^1^ PE + PC + PS + PI = 100%.

The presented results show that each type of cell differs significantly in terms of the level of phospholipids per 10^6^ cells. However, the attempt to compare the obtained results with the data from the literature turned out to be very difficult. Most authors provide their data as a percentage of the total phospholipids, and absolute values are usually expressed as the amount of phospholipid per 10^6^ cells or otherwise the tissue mass are not available. In the case of prostate cells, the comparison of the percentage content of individual GPLs with the results of some authors [45,46] showed a large convergence, but there were considerable differences with the results of other authors [47]. It was presumed that, apart from the various methods of determination of GPL, the underlying cause was the various lipid isolation procedures applied, which was also demonstrated in this study. Application of ultrasound-assisted extraction, an increase in the number of extraction cycles and the modification of the solvent composition at the last extraction step made it possible to recover much more GPLs from biological material than was initially expected. It was also not insignificant that in each cell culture, even established cell lines may present different phenotypes, including the level of phospholipid synthesis, depending on the conditions of its cultivation. No data about the GPL profile in skin cells, such as BJ or HTB 140, were found in the available literature. 

Our results revealed that compared to normal cells, neoplastic cells are characterized by a higher absolute content of PE and PC per 10^6^ cells, although the difference in the percentage content did not correspond to this. In turn, the percentage of PS and PI in both pairs of cells was lower in neoplastic than in normal cells; however, in the case of PNT1A and LNCaP, the difference was not statistically significant. The results obtained in this study do not contradict the reports of other authors, who indicate that neoplastic cells show an increased level of lipids including phospholipids. Changes in the proportion between GPL fractions or in the composition of fatty acids were also found [48,49,50,51,52]. The aforementioned studies mainly considered cancers of the breast, cervix or brain tissue or leukemias. In the case of epithelial cells of the mammary gland, attention has been paid to the differences in the contents of PC and the metabolically related PE [48,50,52], and the statistical significance of the differences found was related to the cancer stage or the degree of malignancy. This phenomenon was explained by an increase in the synthesis of building blocks of tumor cells related to the proliferation rate and the demand for high-energy phosphorus-containing compounds, which are similar to the cells of rapidly developing tissues.

Changes in the phospholipid profile of cancer cells compared to normal ones may potentially indicate an underlying metabolic pathways or processes disturbed by neoplastic transformation. Therefore, phospholipid profiling may have a diagnostic value in the identification of metabolic disorders associated with neoplastic changes in given cells.

## 3. Materials and Methods

### 3.1. Reagents and Reference Materials

Methanol (MeOH), 2-propanol (IPA), n-hexane (Hex), chloroform (CHCl_3_)—all HPLC grade—and acetic acid 100% (CH_3_COOH) were purchased from Merck (Darmstadt, Germany). Triethylamine (TEA) was purchased from Sigma-Aldrich (St. Louis, MO, USA). Ready-to-use sodium chloride 0.9% solution (NaCl) was manufactured by Baxter (Kutno, Poland).

Reference materials, namely, L-α-phosphatidylcholine from egg yolk (PC), L-α-phosphatidyl-ethanolamine from bovine brain (PE), L-α-phosphatidyl-L-serine from bovine brain (PS), L-α-lysophosphatidylcholine from chicken egg (LPC), L-α-phosphatidylinositol from bovine liver (PI) and L-α-phosphatidic acid from egg yolk (PA) were purchased from Sigma-Aldrich (St. Louis, MO, USA). Cardiolipin from bovine heart (CL), sphingomyelin from porcine brain (SM), L-α-phosphatidyglycerol from chicken egg (PG) and L-α-lysophosphatidylserine from porcine brain (LPS) were from Avanti Polar Lipids (Alabaster, AL, USA). All reference standards were dissolved in a CHCl_3_:MeOH (2:1 v/v) mixture. For quantitative purposes, stock solutions of PE, PC, PS and PI were prepared at concentrations of 17 (PE), 20 (PC), 3.3 (PS) and 1.9 mg/mL (PI), which were finally diluted with the same solvent to obtain calibration curves of concentrations in the ranges given in Table 3. Other phospholipids (i.e., PA, PG, CL, SM, LPC and LPS) were prepared with the same solvents at a concentration of approximately 1–2.5 mg/mL for the identification of minor peaks visible in the chromatograms of cell extracts. The system suitability and the stability of the detector response were both monitored with a 1 mg/mL solution of soybean lecithin (brand name Asolectin, Sigma-Aldrich, St. Louis, MO, USA) containing PC, PE, PI and LPC. A cholic acid solution with a concentration of 0.66 mg/mL was used as an internal standard (IS).

Highly purified deionized water was prepared using a Milli-Q Direct 8 water purification system (Merck-Millipore, Billerica, MA, USA).

The following materials for cell culturing were used: RPMI 1640 medium with L-glutamine, EMEM medium with L-glutamine (Lonza, Verviers, Belgium) and DMEM medium with L-glutamine, glucose and sodium pyruvate (Biowest, Nuaillé, France). Fetal bovine serum (FBS), a mixture of antibiotics (Penicillin–Streptomycin–Amphotericin B) and trypsin–EDTA solution were all purchased from Sigma-Aldrich (St. Louis, MO, USA). Phosphate-buffered saline (PBS) without Ca^2+^ and Mg^2+^ ions was obtained from the Institute of Immunology and Experimental Therapy (Wroclaw, Poland). Growth media enriching solutions with 45% glucose, 1 M HEPES buffer and 100 mM sodium pyruvate were purchased from Sigma-Aldrich (Gillingham, UK). 

### 3.2. Cell Lines and Cultivation Conditions

The method of phospholipid determination in biological material was developed using normal PNT1A (ECACC, Salisbury, UK) and neoplastic LNCaP (ATCC, Manassas, VA, USA) as established prostate cell lines. Additionally, the applicability of the developed method was verified with normal fibroblast cells (BJ) and melanoma cells (HTB 140) (ATCC, Manassas, VA, USA).

The cell lines were cultured at 37 °C with 5% CO_2_ (incubator Jouan IGO 150 Cell Life CO_2_, Jouan Inc., Winchester, VA, USA) using dedicated growing media containing 10% FBS and 1% antibiotics mixture. The RPMI 1640 medium with L-glutamine was used for PNT1A and LNCaP cells, EMEM medium with L-glutamine for BJ cells and DMEM medium with L-glutamine, glucose and sodium pyruvate for HTB 140 cells. In the case of the LNCaP cell line, the medium was additionally enriched with HEPES, sodium pyruvate and glucose to obtain a final concentration of 10 mM, 1 mM and 4.5 g/L, respectively, in accordance with ATCC recommendations. Cells were grown in 75 cm^2^ cell culture flasks (NUNC, Roskilde, Denmark). 

The size of the inoculum for each type of cell was optimized in such a way that after 3–4 days of harvesting, approximately 80% monolayer coverage of the bottle surface was obtained. Cells were observed using an ECLIPSE TS-100 inverted microscope (Nikon Corporation, Tokyo, Japan), taking note of the confluence and morphology of the cells.

After 3–4 days of incubation, a homogeneous suspension of cells was prepared by detaching them from the surface after treatment with trypsin–EDTA solution for 2–10 min and incubation at 37 °C and 5% CO_2_ followed by resuspending them in the culture medium. The number of cells in 1 mL of medium was determined with a Coulter Z2 particle counter (Beckman Coulter Corporation, Miami, FL, USA).

For the determination of phospholipids, the cell suspension was centrifuged in 15 mL centrifuge tubes (Nest, Jiangsu, China) for approximately 8 min at 405× *g* (Sigma 3K15, Osterode am Harz, Germany). The obtained cell pellet was washed twice with 10 mL of cold PBS (approximately 4 °C). After the PBS solution was removed, the cell pellet was suspended in a 0.9% NaCl solution in a volume appropriate to obtain approximately 50 × 10^6^ cells/mL. The biological material was stored at −70 °C until analysis (MDF-U3286S Sanyo low-temperature freezer, Osaka, Japan).

### 3.3. Sample Preparation for Phospholipids Assay

Phospholipids from cell membranes were isolated using ultrasound-assisted extraction (UAE) applying elements of the Folch method [25] and the Bligh and Dyer method [26,27]. For this purpose, 1 mL of 0.9% NaCl solution was added to 1 mL of cell suspension in 0.9% NaCl solution, and cells were disrupted with ultrasound (20 kHz, 200 W, cycle 50%, amplitude output 30%) for 10 s (Sonifier 250 Branson Ultrasonics, Danbury, CT, USA). A cold mixture of 2:1 *v*/*v* CHCl_3_:MeOH (2 mL) was added and sonicated again for 10 s, vigorously mixed, and kept on ice for approximately 10 min. After incubation, the sample was centrifuged for 10 min at 3220× *g* at 8 °C (Haereus Megafuge 1.0R, Thermo Scientific, Walthman, MA, USA). The separated, lower organic layer was transferred to a separate tube. The extraction was repeated with another 2 mL of cold CHCl_3_:MeOH (2:1 *v*/*v*), followed by a third extraction with 2 mL of cold CHCl_3_:MeOH (1:1 *v*/*v*). The combined organic phases were evaporated under nitrogen. The residue was dissolved in 0.2 mL of CHCl_3_:MeOH (2:1 *v*/*v*) containing cholic acid at a concentration of approximately 0.66 mg/mL as an internal standard.

### 3.4. Chromatographic Conditions

The chromatographic separation was performed using the Dionex Ultimate 3000 HPLC system (Dionex, Sunnyvale, CA, USA). The system consisted of a dual low-pressure gradient pump DGP-3600A, allowing a ternary gradient formation, a degasser SRD-3600, an autosampler WPS-3000TSL and a column oven TCC-3200. The separations were carried out with a normal stationary-phase LiChrospher Diol 250 × 4.0 mm, 5 µm column, supplied by Merck (Darmstadt, Germany). The Chromeleon software v.6.8 was used for data acquisition and analysis.

During chromatographic runs, samples were kept at 10 °C, and the injection volume was 20 μL. Peaks were measured by charged aerosol detector (Corona CAD) from ESA Bioscience, Inc. (Chelmsford, MA, USA). Chromatography was performed in a binary gradient mode. Eluent A was a mixture consisting of Hex:IPA:CH_3_COOH:TEA (815:170:15:0.8 *v*/*v*/*v*/*v*) and eluent B consisted of IPA:H_2_O:CH_3_COOH:TEA (840:140:15:0.8 *v*/*v*/*v*/*v*). The mobile phase flow was 0.65 mL/min, and the column was thermostated at 50 °C. The gradient time program used for phospholipids separation is summarized in Table 4. The total run time was 61 min, including 18 min for column re-equilibration. CAD detection was performed with a nitrogen pressure of 35 psi, a data collection rate of 10 Hz and with a ”medium filter” setting.

### 3.5. Calculations and Statistical Analysis

Due to the observed certain instability of the CAD signal during long-term analyses (sequences), the normalization of the peak areas of the substances tested in relation to the peak of internal standard was applied. The content of GPL in cell extracts was calculated by comparing the normalized analyte signal with the standard curve, assuming a power relationship between the substance concentration and the CAD signal [34]. To linearize the data, a transformation using the PFV (power function value) was used, the value of which was determined according to [53] and optimized based on minimizing the RSD of response factors for the concentration range adjusted to the analyte signal in samples. The calculations were carried out using Excel sheets from MS Office 2010 software. The results are expressed in mg/mL of the extract or in nmol/10^6^ cells, assuming the molar masses of the individual GPLs according to Avanti Polar Lipids [54], i.e., PE—756.3; PC—786.6; PS—825.0; PI—902.1 g/mol. The Excel Data analysis pack add-on was used to evaluate the equivalence of variance (F-Snedecor’s test) and the equivalence of means (Student’s *t*-test) between data groups. In all tests, the significance level was set at α = 0.05.

## 4. Conclusions

The conducted research allowed us to conclude that the developed method of HPLC with CAD detection enables the separation and quantification of the phospholipids: PE, PC, PS and PI in biological material at the required concentration level. 

Based on the historical methods of Folch and Bligh–Dyer, an improved method was developed for isolating phospholipid from cells using UAE and three-fold extraction with solvents with different proportions of chloroform and methanol, resulting in a significant increase in the efficiency in relation to the tested substances. The use of the internal standard method allowed us to remove the drawbacks related to the instability of the CAD detector response and to obtain reproducible results. The transformation of the data using PFV, allowed for the linearization of the calibration curve and, thus, the calculation of the content of the test substances was possible in a wide concentration range. The results of the validation performed for analytical range, precision, intermediate precision and accuracy showed that the analytical procedure is suitable for assessing the phospholipid content in cell extracts. The developed method may be a useful tool for profiling phospholipids in cell membranes or other biological materials. The assessment of GPL level, using the developed method, may contribute to clarifying the mechanisms underlying various diseases, including neoplastic processes, and be used for diagnostic purposes.

## Figures and Tables

**Table 1:** Chromatographic performance assessed during the robustness test. Column No. 1 was used in current study.

Column		Resolution between		S/N forPS
No.	Description, Dimensions	PE/PC	PI/LPC	
1	Lichrospher Diol 100 250 × 4 mm, 5 μm, batch A	2.4	3.8	99.1
2	Lichrospher Diol 100 250 × 4 mm, 5 μm, batch B	3.7	4.5	86.0
3	Lichrosorb Diol 250 × 4 mm, 5 μm	2.4	3.2	89.3
4	Supelcosil LC-Fuji-Diol 250 × 4.6 mm, 5 μm	3.2	4.8	9.8

**Table 2:** Validation results of the developed method.

Phospholipid	Analytical Range (mg/mL)	Determination Coefficient *R*^2^ for Linearized Data	S/N for the Lowest Concentration of Analytical Range	Concentration at Precision Test (mg/mL)	Precision (n = 6), RSD (%)	Intermediate Precision (n = 12), RSD (%)	Recovery (%)
PE	0.40–9.40	0.9968 (n = 10)	220	2.4	9.03%	8.10%	104.5%
PC	0.20–10.0	0.9963 (n = 12)	620	3.1	3.52%	5.90%	98.9%
PS	0.04–1.70	0.9969 (n = 12)	250	0.3	4.32%	5.18%	108.0%
PI	0.07–1.75	0.9978 (n = 10)	90	0.5	5.01%	8.46%	96.3%

n = number of calibration points or size of the group.

**Table 4:** Optimized gradient time program. Dwell volume: approximately 1.5 mL.

Time (min)	Solvent A (%)	Solvent B (%)
0	95	5
3	95	5
35	50	50
37	20	80
41	20	80
43	95	5
61	stop	

## Data Availability

Not applicable.
